# Accelerated risk of renal disease progression in pre-ESRD patients with proton pump inhibitors use: a nationwide population-based study

**DOI:** 10.1186/s12882-024-03867-6

**Published:** 2024-12-23

**Authors:** Chien-Huei Huang, Chih-Jung Tsai, Chien-Chou Su, Chi-Tai Yen, Ju-Ling Chen, Ching-Lan Cheng

**Affiliations:** 1 Department of Pharmacy, College of Medicine, National Cheng Kung University Hospital, National Cheng Kung University, Tainan, Taiwan; 2 School of Pharmacy, Institute of Clinical Pharmacy and Pharmaceutical Sciences, College of Medicine, National Cheng Kung University, No. 1 University Road, Tainan City, 701 Taiwan; 3 Department of General Medicine, Chi-Mei Medical Center, Tainan, Taiwan; 4 Department of Nephrology, Ministry of Health and Welfare, Tainan Hospital, Tainan, Taiwan; 5 Health Outcome Research Center, National Cheng Kung University, Tainan, Taiwan; 6 Clinical Innovation and Research Center, National Cheng Kung University Hospital, No. 138 Sheng Li Road, Tainan, Taiwan

**Keywords:** Proton pump inhibitors, H2 blocker, pre-ESRD, Nephrotoxicity, Asia

## Abstract

**Background:**

Although Proton pump inhibitors (PPIs) were mostly prescribed for gastrointestinal (GI) disease widely, there were numerous studies about PPIs and adverse renal outcome. Most evidence was to evaluate the risk of PPIs in patients with normal renal function and in the absence of the moderate to advanced chronic kidney disease (CKD). This study focuses on the accelerated progression of renal function following proton pump inhibitors (PPIs) use, and the increased risks of acute kidney injury (AKI) among moderate to advanced CKD (pre-ESRD) patients.

**Patients and methods:**

A retrospective cohort study was conducted by including adult patients with chronic kidney disease (CKD) stages 3b to 5 who initiated PPI or H2 blocker (H2B) therapy between 2011 and 2018. The risk of renal events was assessed using the Cox proportional hazard model to estimate the adjusted hazard ratio (HR) and 95% confidence interval (CI). Sensitivity analyses were performed, including propensity score matching, as-treated analysis, and subgroup analysis.

**Results:**

The cohort comprised 83,432 pre-ESRD patients, with 5,138 treated with H2B and 1,051 with PPIs. The progression to ESRD was significantly more likely in patients using PPIs compared to those using H2B (adjusted HR, 1.495; 95% CI: 1.198–1.867). Specifically, omeprazole (adjusted HR, 1.784; 95% CI: 1.079–2.951) and esomeprazole (adjusted HR, 1.847; 95% CI: 1.332–2.561) were associated with a notably higher risk of ESRD and AKI.

**Conclusions:**

The study highlights the significance of the accelerated renal risk, especially for moderate to advanced CKD patients, when prescribing PPIs and to implicate the clinicians prescribed PPIs and H2B in pre-ESRD patients.

**Supplementary Information:**

The online version contains supplementary material available at 10.1186/s12882-024-03867-6.

## Key message

1. Proton pump inhibitors, particularly omeprazole and esomeprazole, are associated with a higher risk of progressing to end-stage renal disease (ESRD) and acute kidney injury (AKI) in pre-ESRD patients.

2. The study underscores the influence of the progression of moderate to advanced chronic kidney disease following PPIs compared H2Bs treatment, highlighting the importance risk factors in prescribing these medications.

3. The findings showed the expedited risk of progression of kidney disease, especially for moderate to late-stage chronic kidney disease (CKD) patients and provided implications for prescribing and de-prescribing PPIs in order to minimize renal risks.

Plain Summary:

The findings showed that proton pump inhibitors users had a higher risk of progression of kidney disease, especially for moderate to late-stage chronic kidney disease patients.

**Supplementary Information:**

The online version contains supplementary material available at 10.1186/s12882-024-03867-6.

## Introduction

Proton pump inhibitors (PPIs) are established as the leading medications for gastric acid suppression, and have a crucial role in treating peptic ulcer disease, gastroesophageal reflux disease (GERD), and in eradicating Helicobacter pylori. Their significant clinical use is well-documented [1]. However, recent studies have raised concerns about the long-term use of PPIs, particularly the potential risk of adverse renal outcomes. These range from minor issues such as hypomagnesemia to serious conditions such as acute kidney injury (AKI), chronic kidney disease (CKD), and even the risk of progression to end-stage renal disease (ESRD) [1–5]. The underlying mechanisms of PPI-induced renal dysfunction, potentially involving a dose-response relationship, are an area of ongoing research [6, 7].

The metabolic processing of PPIs, especially omeprazole and esomeprazole, which predominantly rely on the Cytochrome P450 2C19 (*CYP2C19*) enzyme, is a key issue. Studies indicate a significant variability in *CYP2C19* activity among different ethnic populations, with Asians often exhibiting a slower metabolic rate compared to Western populations [8]. This variation suggests that Asian populations may be more likely to accelerate adverse renal outcomes, when treated with *CYP2C19*-metabolized PPIs.

Taiwan, despite implementing a pre-ESRD pay-for-performance program in 2006 to enhance care for CKD stages 3b to 5 patients, continues to have one of the highest global ESRD prevalence [9]. This alarming statistic underscores the importance of region-specific research on potential renal risks associated with PPIs. Current research in Taiwan on PPI-related renal risks primarily comprises case-control studies [10, 11]. While these studies have been valuable in suggesting potential associations, their design limits the ability to draw definitive conclusions. This limitation highlights the need for further research using more rigorous methodologies to better understand the potential effects of PPI usage on renal outcomes.

Our study investigates to expand upon these findings by exploring the relationship between PPI usage and the risk of AKI and progression to ESRD specifically in the pre-ESRD patient in Taiwan. We focus particularly on PPIs, exploring whether these medications accelerated e the risk of renal function progression in pre-ESRD patients. By examining the implications of PPI usage in a broader and more extended context, this research aims to provide valuable insights into renal health outcomes. These findings will contribute to more informed clinical decisions in the prescription of PPIs or H2 blockers (H2B) especially in moderate to advanced CKD patients, particularly in these population in pre-ESRD program in Taiwan.

## Patients and methods

### Study design

Our study was a retrospective, nationwide population-based cohort study conducted in Taiwan. It aimed to estimate the risk of acute kidney injury (AKI) and progression to end-stage renal disease (ESRD) among adult pre-ESRD patients (CKD stage 3b to 5) associated with the use of proton pump inhibitors (PPIs), in comparison to the use of H2 blockers (H2B). The study received approval from the institutional review board of National Cheng Kung University Hospital, Tainan, Taiwan (IRB no.A-EX-108-013).

### Database

This study utilized the National Health Insurance Research Database (NHIRD), provided by Taiwan’s Ministry of Health and Welfare, encompassing comprehensive medical claims from January 1, 2011, to December 31, 2018. The database included both inpatient and outpatient records, detailing disease diagnosis, prescription drugs, medical procedures, and surgeries. All healthcare service providers in Taiwan are mandated to submit diagnosis information, using the International Classification of Disease-Clinical Modification, ninth and tenth revisions (ICD-9-CM and ICD-10), along with all related service claims, for processing by the National Health Insurance. Validation studies of diagnoses in the NHIRD have shown a high positive predictive value for major diseases, making it useful for long-term follow-up observational studies [12, 13]. 

### Subjects

**Fig. 1:**
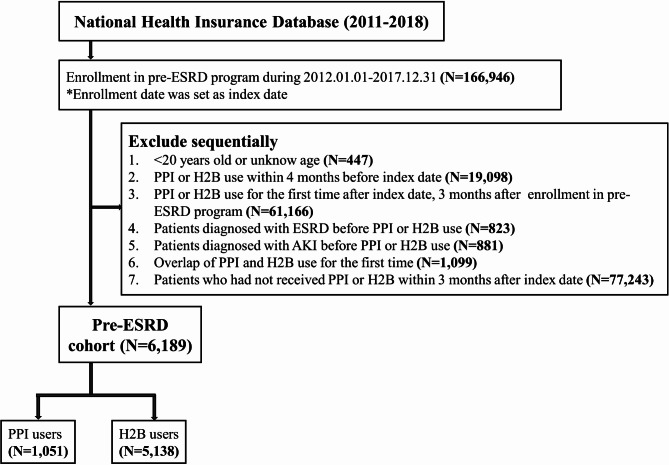
Flowchart of study inclusion. From 2012 to 2017, 83,432 pre-ESRD patients were identified. Of these, we identified 5,138 (6.2%) new histamine-2 receptor blocker (H2B) users and 1,051 (1.3%) new proton pump inhibitor (PPI) users

This study focused on adult patients (≥ 20 years old) enrolled in Taiwan’s pre-ESRD program from January 1, 2012, to December 31, 2017. The pre-ESRD program has been a part of the National Health Insurance (NHI) reimbursement scheme since January 1, 2012. Enrollment in the program was identified using specific reimbursement codes (P3402C to P3409C) found in the ambulatory care order details of the patients (detailed in Table S1). The pre-ESRD program enrolled the patients with stage 3b-5 chronic kidney disease and received multidisciplinary care regularly. The index date for each patient was set as the date of their enrollment in the pre-ESRD program. Patients who initiated PPIs or H2Bs within four months prior to the index date were excluded. This exclusion criteria were based on the NHI payment guidelines in Taiwan, which stipulate a minimum four-month wash-out period for antacid therapy, except for a two-week period in cases of Helicobacter pylori eradication. Additionally, we excluded patients who had a prior diagnosis of ESRD or AKI before the index date and those who were simultaneously prescribed both PPIs and H2Bs. For the study, we included only those patients who were prescribed PPIs or H2Bs within three months following their enrollment in the pre-ESRD program to reduce information bias because of patients enrolled in the pre-ESRD program are regularly followed up by doctors and closely monitored. (Fig. 1). The “three-month” time frame was chosen because the validity period for chronic continuous prescriptions in Taiwan is three months.

### Study exposure and follow-up duration

Our study adopted a first-exposure-carried-forward approach, akin to an “intention-to-treat” analyses, without censoring for discontinuation or switching of medications. Patients with at least one prescription during the one-year follow-up period were classified as PPI or H2B users. The cohort included new users of PPIs, forming the treatment group, and new users of H2Bs as the active comparator. We tracked the new users until the occurrence of any study outcome follow-up one year from the start of the therapy, death, or until the end of 2018, whichever came first. The reason for limiting the follow-up to one year is based on the restrictions for PPI prescriptions. According to reimbursement criteria, patients with GI bleeding or active ulcers confirmed by endoscopy can be prescribed PPIs for up to one year.

### Study covariates

At the index date, age and sex of the patients were recorded. We assessed comorbidities from both inpatient and outpatient claims for three years preceding the index date. These comorbidities encompassed indications for acid-suppression therapy such as GERD, gastrointestinal bleeding, peptic ulcer disease, esophageal varices, and Helicobacter pylori infection, along with other health conditions like cerebrovascular disease, peripheral artery disease, cardiovascular disease, hyperlipidemia, hypertension, diabetes mellitus, chronic obstructive pulmonary disease (COPD), dementia, cancer, and viral hepatitis. The details of these comorbidities are detailed in Table S2. Co-medication use for the six months prior to the index date was also recorded, following the anatomical therapeutic chemical classification system. This included non-steroid anti-inflammatory drugs (NSAIDs), renin-angiotensin-aldosterone system (RAAS) inhibitors, calcineurin inhibitors, diuretics, antivirals, antibiotics, calcium channel blockers (CCBs), β-blockers, antithrombotic agents, and statins.

### Study outcomes

The primary outcome of this study was the progression to ESRD, identified using ICD-9-CM code 585 or ICD-10 code N18.6 from the Registry for Catastrophic Illness Patients. This certificate is issued only after a physician’s diagnosis and a formal review by the Bureau of National Health Insurance in Taiwan. The catastrophic illness certificate entitles patients to subsidies and waivers for outpatient and inpatient copayment. The secondary outcome was AKI, defined by ICD-9-CM code 584 or ICD-10 code N17. The codes used to identify AKI were validated in a study conducted by NSARF (National Taiwan University Hospital Study Group on Acute Renal Failure), with a positive predictive value of 98.5% and a negative predictive value of 74.0% [14]. Additionally, subgroup analyses were conducted to assess the risks associated with individual PPIs.

### Statistical analysis

Descriptive analyses were presented as mean ± SD or percentage. Categorical variables were analyzed using the Chi-squared test, while continuous variables were assessed with Student’s t-test. Kaplan–Meier survival curves were constructed to compare time-to-event outcomes using the log-rank test. Hazard ratios (HRs) with 95% confidence intervals (CIs) were calculated using univariate and multivariate Cox proportional hazards models. The multivariate models for potential adjusted for potential confounders, including covariates of baseline characteristics. A *P* < 0.05 was considered statistically significant. All statistical analyses were conducted using SAS version 9.4 (SAS Institute, Cary, NC, USA).

### Sensitivity analyses

To evaluate the robustness of our results, several sensitivity analyses were performed. Firstly, a propensity score analysis was conducted to minimize selection bias arising from clinical characteristic differences between groups. Multivariate logistic regression analysis, based on relevant covariates, was used to compute the propensity scores for receiving PPI or H2B. Patients were then matched by propensity score with a 1:1 ratio using the Greedy technique. Secondly, an as-treated design was employed to assess the risk of AKI and ESRD based on the actual use of PPIs and H2B during the one-year follow-up period. Thirdly, covariates with statistically significant P-values were selected to stratified patients into subgroups for further comparative analyses. Lastly, we used the E-value to explore the effect of unmeasured confounding related to CKD stage.

## Results

### Baseline characteristics

Between 2012 and 2017, a total of 83,432 pre-ESRD patients were identified in our study (Fig. 1). Among these, 77,243 patients (92.6%) did not receive treatment with either PPIs or H2Bs, 5,138 patients (6.2%) were treated with H2Bs, and 1,051 patients (1.3%) received PPIs. The baseline characteristics of the patients in the PPI and H2B cohort are presented in Table 1. (non-users presented in Table S4).

**Table 1:** Baseline characteristics of Pre-ESRD subjects by the medication group

	H2B users (*n* = 5,138)		PPI users (*n* = 1,051)		*P* Value
**Age**, **mean (SD) y**	69.7	(13.3)	71.0	(12.9)	0.0042
**Male**, **n (%)**	2,855	(55.6)	646	(61.5)	0.0004
**Comorbidities**, **n (%)**					
GERD	441	(8.6)	107	(10.2)	0.0967
GI hemorrhage	185	(3.6)	59	(5.6)	0.0022
Peptic ulcer disease	798	(15.5)	164	(15.6)	0.9526
HP infection	30	(0.6)	7	(0.7)	0.7529
Cerebrovascular disease	850	(16.5)	225	(21.4)	0.0001
Peripheral artery disease	289	(5.6)	56	(5.3)	0.7027
Cardiovascular disease	3,747	(72.9)	728	(69.3)	0.0157
Hyperlipidemia	2,218	(43.2)	404	(38.4)	0.0047
Hypertension	4,244	(82.6)	877	(83.4)	0.5094
Diabetes mellitus	2,879	(56.0)	601	(57.2)	0.4935
COPD	627	(12.2)	129	(12.3)	0.9491
Dementia	273	(5.3)	68	(6.5)	0.1343
Cancer	545	(10.6)	138	(13.1)	0.0174
Viral hepatitis	231	(4.5)	48	(4.6)	0.9193
**Medication history**,** n (%)**					
NSAIDs	2,930	(57.0)	546	(52.0)	0.0025
RAAS inhibitors	3,749	(73.0)	724	(68.9)	0.0071
Calcineurin inhibitors	27	(0.5)	8	(0.8)	0.3532
Diuretics	2,416	(47.0)	582	(55.4)	< 0.0001
Antivirals	72	(1.4)	15	(1.4)	0.9482
Antibiotics	2,188	(42.6)	446	(42.4)	0.9291
CCBs	2,953	(57.5)	648	(61.7)	0.0123
β-blockers	2,239	(43.6)	473	(45.0)	0.3954
Antithrombotics	2,704	(52.6)	547	(52.0)	0.7307
Statins	2,124	(41.3)	404	(38.4)	0.0814

H2B, histamine H2-blockers; PPI, proton pump inhibitor; SD, standard deviation; GERD, gastroesophageal reflux disease; GI, gastrointestinal; HP, *Helicobacter pylori*; COPD, chronic obstructive pulmonary disease; NSAIDs, non-steroid anti-inflammatory drugs; RAAS, renin-angiotensin-aldosterone system; CCBs, calcium channel blockers; ESRD; end-stage renal disease

A comparison of these groups revealed that patients in the H2B cohort were generally younger than those in the PPI cohort. Additionally, the prevalence of various comorbidities and medication history was found to be higher in the PPI cohort compared to the H2B cohort. These comorbidities included GERD, gastrointestinal hemorrhage, peptic ulcer disease, Helicobacter pylori infection, cerebrovascular disease, cardiovascular disease, hypertension, diabetes mellitus, COPD, dementia, hyperlipidemia, cancer, and viral hepatitis. The medication history of the PPI cohort more frequently involved the use of NSAIDs, RAAS inhibitors, calcineurin inhibitors, diuretics, CCBs and β-blockers.

### Clinical outcomes

**Fig. 2:**
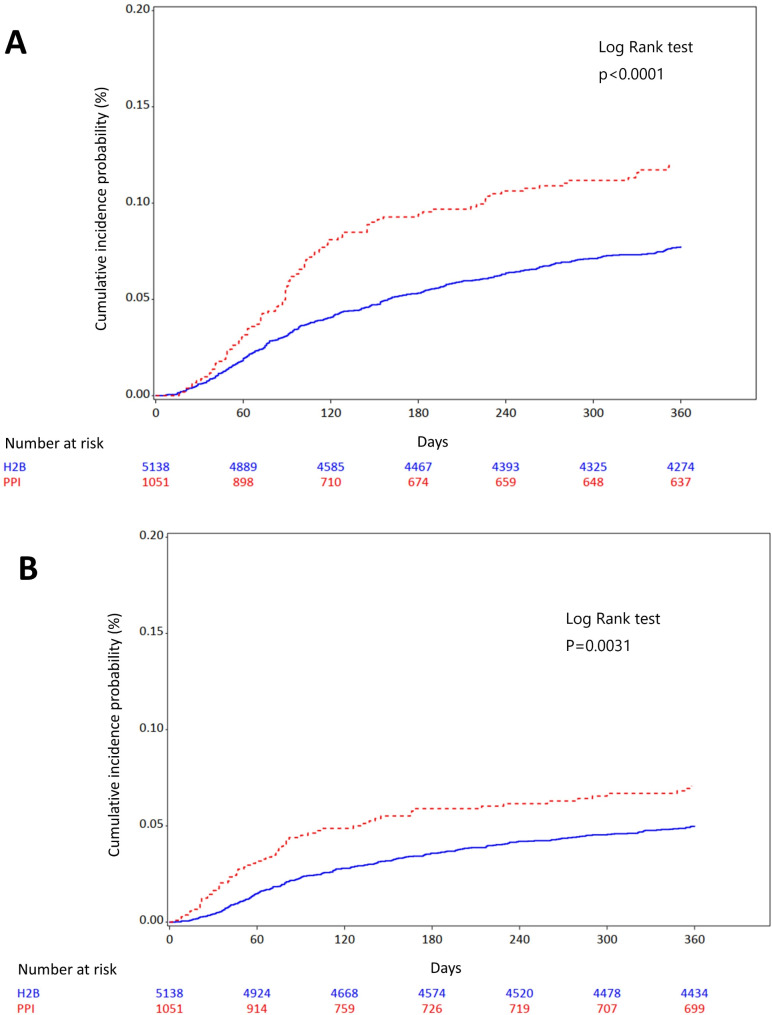
Kaplan-Meier curve for the risk of (**A**) progression to ESRD and (**B**) AKI before the propensity-score matched cohorts treated with H2B (solid line) or PPI (dotted line) in pre-ESRD patients. Kaplan–Meier curve illustrating the cumulative incidence of (**A**) progression to ESRD and (**B**) AKI in per-ESRD patients newly prescribed proton pump inhibitors (PPIs) and histamine-2 receptor blockers (H2B). We followed up new users of PPI or H2B to be on therapy until the censor points, which are the first occurrence of any study outcome within 1 year, death, end of year 2018, or follow up duration until 1 year, whichever came first. A visual inspection suggests that risk of progression to ESRD and AKI seems to be higher in PPI users, compared to H2B users. AKI, acute kidney injury; ESRD, end stage renal disease

In our intention-to-treat analysis, the primary outcome, progression to ESRD, occurred in 9.71% of PPI users compared to 7.36% in H2B users during about 1 year follow-up (Table 2). The adjusted HR for this outcome was 1.495 (95% CI: 1.198–1.867, Fig. 2A). Similarly, the secondary outcome, AKI, was observed in 6.18% of PPI users and 4.81% of H2B users, with an adjusted HR of 1.395 (95% CI: 1.058–1.840, Fig. 2B). Specific analysis of individual PPIs showed that both omeprazole and esomeprazole were significantly associated with a higher risk of progression to ESRD and AKI, with adjusted HRs of 1.784 (95% CI: 1.079–2.951) and 1.847 (95% CI: 1.332–2.561). Conversely, rabeprazole was associated with a potentially lower risk of progression to ESRD and AKI (Table 3).

**Table 2:** Association between progression to ESRD and AKI, comparing PPI and H2B users (primary analysis and sensitivity analysis)

	PPI users (*n* = 1,051)	H2B users (*n* = 5,138)	Crude HR (95% CI)	Adjusted HR^a^ (95% CI)
**Primary analysis (Intention-to-treat design)**				
Progression to ESRD	102 (9.71)	378 (7.36)	1.613 (1.296–2.007)	1.495 (1.198–1.867)
AKI	65 (6.18)	247(4.81)	1.506 (1.146–1.980)	1.395 (1.058–1.840)
**Sensitivity analysis (PS 1:1 matching design**, ***n*** **=** **1051 in both users)**				
Progression to ESRD	102 (9.71)	89 (8.47)	-	1.359 (1.023–1.807)
AKI	65 (6.18)	39 (3.71)	-	1.903 (1.279–2.831)
**Sensitivity analysis (As-treated design)**				
Progression to ESRD	36 (3.43)	93 (1.81)	2.300 (1.571–3.370)	2.184 (1.477–3.229)
AKI	39 (3.71)	99 (1.93)	2.144 (1.452–3.166)	1.909 (1.284–2.837)

PS: Propensity score; ESRD, end-stage renal disease; AKI, acute kidney injury; HR, hazard ratio; CI, confidence interval; H2B, H2 blocker

^a^Adjusted variables: age, gender, Comorbidities (GERD, GI hemorrhage, Peptic ulcer disease, HP infection, Cerebrovascular disease, Peripheral artery disease, Cardiovascular disease, Hyperlipidemia, Hypertension, Diabetes mellitus, COPD, Dementia, Cancer, Viral hepatitis), Medication history (NSAIDs, RAAS inhibitors, Calcineurin inhibitors, Diuretics, Antivirals, Antibiotics, CCBs, β-blockers, Antithrombotics, Statins)

**Table 3:** The effect of individual PPI use on the risk of ESRD (vs. H2B, event rate = 7.36%) and AKI (vs. H2B, event rate = 4.81%)

Individual PPI	*N*	Events	(Event rate, %)	Adjusted HR^a^ (95% CI)	
**Progression to ESRD**					
Dexlansoprazole	23	3	(13.04)	1.89	(0.60–5.95)
Omeprazole	132	16	(12.12)	1.78	(1.08–2.95)*
Esomeprazole	390	41	(10.51)	1.85	(1.33–2.56)*
Lansoprazole	160	16	(10.00)	1.29	(0.78–2.13)
Pantoprazole	289	24	(8.30)	1.19	(0.78–1.80)
Rabeprazole	57	0	(0.00)	0.58	(0.14–2.32)
**AKI**					
Omeprazole	132	11	(8.33)	1.83	(1.00-3.36)
Pantoprazole	289	16	(5.54)	1.24	(0.75–2.07)
Lansoprazole	160	9	(5.63)	1.12	(0.58–2.19)
Rabeprazole	57	0	(0.00)	-	-
Esomeprazole	390	29	(7.44)	1.84	(1.25–2.71)
Dexlansoprazole	23	0	(0.00)	0.00	-

*p < 0.05

ESRD, end-stage renal disease; AKI, acute kidney injury; HR, hazard ratio; CI, confidence interval; H2B, H2 blocker; PPI, proton pump inhibitors

^a^Adjusted variables: age, gender, Comorbidities (GERD, GI hemorrhage, Peptic ulcer disease, HP infection, Cerebrovascular disease, Peripheral artery disease, Cardiovascular disease, Hyperlipidemia, Hypertension, Diabetes mellitus, COPD, Dementia, Cancer, Viral hepatitis), Medication history (NSAIDs, RAAS inhibitors, Calcineurin inhibitors, Diuretics, Antivirals, Antibiotics, CCBs, β-blockers, Antithrombotics, Statins)

The first sensitivity analyses, employing propensity score matching, is presented in Table 2. After matching, a total of 2,102 pre-ESRD patients (1,051 per group) were selected, resulting in more balanced baseline characteristics between groups (Table S3). The density plots of the propensity scores before and after matching are shown in Figure S1. Post-matching, the incidence of ESRD in PPI users was 9.71%, and 8.47% in H2B users, with an adjusted HR of 1.359 (95% CI: 1.023–1.807). For AKI, the incidences were 6.18% in PPI users and 3.71% in H2B users, with an adjusted HR of 1.903 (95% CI: 1.279–2.831).

Under the as-treated design, the incidence of the primary outcome (ESRD) was 3.43% in PPI users and 1.81% in H2B users (adjusted HR: 2.184, 95% CI: 1.477–3.229). The incidence of the secondary outcome (AKI) was 3.71% in PPI users and 1.93% in H2B users (adjusted HR: 1.909, 95% CI: 1.284–2.837) (Table 2).

After adjusting for covariates with statistically significant P-values, our subgroup analyses revealed a consistent trend across various subgroups, as illustrated in Figures S2 and S3.

Supplementary Figure S4 showed that the E-value was 2.36 for the relative risk (RR) of exposure and unmeasured confounding and the RR of unmeasured confounding and disease.

## Discussion

In our comprehensive, nationwide population-based study in Taiwan, we observed a significant association between the use of PPIs and the increased risk of progression to ESRD and AKI, compared to H2B use, in patients with moderate to advanced kidney disease (CKD stage 3b to 5). This association was notably pronounced in PPIs, such as omeprazole and esomeprazole.

Most previous cohort studies [2, 3, 7, 15–21] have identified an association between PPI use and adverse renal outcomes, including incident CKD, ESRD, and AKI, in patients with normal baseline renal function. In contrast, our study specifically focused on patients with moderate to advanced kidney disease (CKD stages 3b to 5). This difference in the study population likely explains why we observed a higher incidence of progression to ESRD compared to previous studies that included patients with normal renal function.

Similar findings were reported by Grant et al. [17] and Liabeuf et al. [19], who examined PPI safety in CKD (estimated Glomerular filtration rate, eGFR < 60 mL/min/1.73m^2^) and employed a new-user study design, but compared to non-PPI use. Grant et al. [17] identified that an association between PPI use and an increased risk of major adverse renal events, including the doubling of creatinine or progression to ESRD. Liabeuf et al. [19] also noted a significant association between PPI use and an elevated risk of ESRD, particularly in new users compared to long-term users, and found a dose–response relationship with PPIs, based on the defined daily dose (DDD).

Our findings contrast with Cholin et al. study [18], which also focused on patients with established CKD and utilized H2B as an active comparator. Although Cholin et al. enrolled patients with CKD (eGFR < 60 mL/min/1.73m^2^), the patients with CKD stage 3a was more than 70% in this study population. Cholin et al. reported that PPI use in a CKD population was not associated with an increased progression to ESRD compared to H2B use or no medication, with cumulative incidence at four years being 2.0%, 1.5%, and 1.6% respectively (*P* = 0.22).

Several key differences between our study and Cholin et al.’s might account for the discrepancy in study findings. Firstly, both our study and Cholin’s demonstrated imbalances between PPI and H2B users in baseline characteristics. To address this in our research, we implemented 1:1 propensity-score matching, aiming to minimize potential biases. Secondly, our study employed a new-user design, which likely reduced the confounding effect by indication. Furthermore, Cholin et al. utilized Fine-Gray subdistribution hazard models to evaluate the relationship between PPI use and ESRD progression, considering death as a competing risk. This approach could potentially lead to an underestimation of the actual incidence. Additionally, the study populations in the research conducted by Cholin et al.’s [18], Grant et al.’s [17] and Liabeuf et al.’s [19] had eGFR less than 60 mL/min/1.73m^2^, while our study focused on a population with eGFR less than 45 mL/min/1.73m^2^ (CKD stage 3b, 4 and 5). According to Annual Report on Kidney Disease in Taiwan, the patients with CKD stage 3b, 4 and 5 was about 36%, 34% and 22%, respectively. It had represented our study population. Another critical difference lies in the ethnic composition of the study cohorts. While Cholin et al.’s research, along with most other previous studies in this field, predominantly involved populations of European and African ancestry, our study primarily included Asian participants. This is relevant because Asian populations are known to have higher frequencies of poor *CYP2C19* metabolizers compared to Western populations [22]. This genetic difference may partly explain why the incidence of progression to ESRD in our study (9.71% in PPI users vs. 7.36% in H2B users) was higher than that observed in Cholin et al.’s study.

The exact mechanisms by which PPIs contribute to renal function deterioration are not fully understood. Previous studies have indicated a link between PPI use and the development of AKI, which may progress to chronicity. However, this association is contested by findings from Xie et al. [23], who reported that AKI does not mediate the relationship between PPI use and the onset of CKD. Several hypotheses have been proposed to explain the potential nephrotoxic effects of PPIs. One theory suggests that PPI use may induce hypomagnesemia or elevate levels of asymmetrical dimethylarginine, both of which are factors associated with the decline in kidney function [16]. Additionally, PPIs are implicated in promoting enteric infections such as *Clostridium difficile*. These infections are thought to result from decreased gastric acidity and alterations in the gut microbiome, as well as the translocation of endotoxins into the circulation, which could contribute to uremic toxicity, inflammation, and the progression of kidney disease [24]. Moreover, the metabolic pathways of most PPIs, with the exception of rabeprazole, involve significant processing (> 80%) by the *CYP2C19* enzyme. Omeprazole and esomeprazole, in particular, act as inhibitors of *CYP2C19* [8]. The drug interaction between PPIs and other nephrotoxic drugs metabolized by *CYP2C19* could further increase the risk of renal function impairment in patients with renal diseases [11]. Additionally, there is a possibility that PPIs, either alone or in combination with other medications, may accumulate in the kidneys, leading to a reduction in renal function.

Moreover, some studies have reported an association between omeprazole and both chronic kidney disease and acute interstitial nephritis. It was also the first PPI-related kidney injury reported as an adverse drug reaction [25]. Cellular and molecular mechanisms have shown that omeprazole directly induces cell death in cultured renal tubular cells, both in vivo and in vitro, through the generation of oxidative stress. It also causes mitochondrial injury, leading to decreased ATP availability and increased oxidative stress, which, in turn, drives cell death [26]. These findings provide biological plausibility to the epidemiological data linking omeprazole with AKI and ESRD.

Furthermore, our subgroup analyses revealed a consistent trend of increased ESRD risk in PPI users compared to H2B users, particularly in patients with advanced chronic renal disease. This effect was more pronounced in patients over 65 years and in females, as shown in Figure S2. Given that GERD more commonly affects women, and the prevalence of reflux esophagitis significantly increases with age in women, especially post-50s [27], prescribing PPIs other than omeprazole or esomeprazole may be a safer alternative for female patients or those over 65, particularly in Asian populations.

To our knowledge, this study represents the first population-based and focused on pre-ESRD population cohort analysis in Asia, a region characterized by a higher prevalence of *CYP2C19* poor metabolizers, to assess the risk of renal-adverse events associated with PPI use in CKD patients. A significant strength of our study is the enrollment of the study population in Taiwan’s pre-ESRD program. This enrollment indicates that the participants were receiving consistent healthcare, allowing us to attribute the observed differences more confidently in ESRD and AKI risk to the use of PPIs and H2B, rather than to variances in healthcare delivery. Additionally, we employed a propensity score method, based on a counterfactual framework, to mitigate confounding factors and enhance the reliability of the association between the drug exposure and the observed outcomes.

Despite its strengths, our study has several limitations. One major constraint is our inability to assess medication compliance and the use of over-the-counter PPIs.

To address this limitation, we selected H2B as an active comparator, recognizing that it is also widely available without prescription. Although over-the-counter PPIs and H2Bs are available in Taiwan, we believe the proportion of over-the-counter (OTC) drugs use in our cohort was very low. This is because there are no restrictions on prescribing H2Bs, and the OTC price of PPIs is generally higher than the insurance copayment. Therefore, patients who receive insurance-covered PPIs are unlikely to purchase PPIs over the counter out of pocket. Approach of active comparator, combined with our as-treated study design, reinforced our findings of a higher risk of CKD progression associated with PPI use. Another limitation is the lack of detailed CKD staging information in the NHIRD. To navigate this issue, we focused on patients enrolled in the pre-ESRD program (also called Pay for performance (P4P) program), who were likely in the moderate to advanced stages of CKD (stages 3b to 5 or presenting with heavy proteinuria). The pre-ESRD program has multidisciplinary care members including nephrologists, nurses and dietitians, providing CKD education knowledge, communication with family, and regular follow-up of the patients’ health status. The care indicators included renal function maintenance, continuous multidisciplinary care, and CKD management and education. To claim the pre-ESRD reimbursement payments, the providers must collect and report data including renal function on the quality indicators to the NHI within 3 months regularly before or after physician visits. Therefore, CKD definition in the pre-ESRD program in Taiwan, not only based on ICD codes but also on accurate report data related to renal function. Additionally, we used the E-value to explore the effect of unmeasured confounding related to CKD stage (E-value was 2.36). If the RR of either exposure to unmeasured confounders or unmeasured confounders to disease exceeds the E-value, our estimate would become null. According to a systematic review, the progression from CKD stages 3–5 to end-stage renal disease (ESRD) had an RR of 1.37 (95% CI 1.17–1.62) [28], with both the point estimate and upper bound being lower than the E-value. Therefore, unmeasured confounders would not invalidate our primary estimation. Moreover, the claims database lacks information on the severity of AKI, making it impossible for us to measure the impact of AKI in PPI or H2B users. Theoretically, greater AKI severity would increase the incidence of ESRD. If AKI severity among H2B users were more severe than in PPI users, the fact that PPI users still exhibited a higher ESRD incidence suggests a heightened risk. Conversely, if AKI severity was greater in PPI users, this would underscore the need for closer attention to the risk of renal progression in PPI users. However, the definitive impact of PPI use on CKD progression requires further exploration, ideally through a prospective cohort study. Lastly, our study’s focus on one-year outcomes means that our findings may not be applicable to longer-term PPI use, necessitating additional research to understand these extended effects.

## Conclusions

Our study in Taiwan has illustrated that the use of PPIs is associated with an increased and accelerated risk of progressing to ESRD and AKI compared to H2B among pre-ESRD patients. This finding is particularly showed the accelerated renal risk than other cohort studies, especially in the pre-ESRD patients. Our subgroup analyses revealed that PPIs like omeprazole and esomeprazole, which exhibit auto-inhibition effects on *CYP2C19*, were associated with higher hazard ratios for ESRD development than other PPIs. Given Taiwan’s status as having the highest global incidence of ESRD, our findings emphasize the necessity for a more personalized approach to PPI use in pre-ESRD populations. This may involve reevaluating the prescription practices for PPIs in high-risk groups, particularly in the late stage CKD and to de-prescribing PPIs to ensure renal safety.

## Electronic supplementary material

Below is the link to the electronic supplementary material.

Supplementary Material 1

Supplementary Material 2

Supplementary Material 3

Supplementary Material 4

Supplementary Material 5

Supplementary Material 6

Supplementary Material 7

Supplementary Material 8

Supplementary Material 9

## Data Availability

No datasets were generated or analysed during the current study.

## Abbreviations

AKI: Acute Kidney Injury
CCBs: Calcium Channel Blockers
CI: Confidence Interval
CKD: Chronic Kidney Disease
COPD: Chronic Obstructive Pulmonary Disease
CYP2C19: Cytochrome P450 2C19
DDD: Defined Daily Dose
eGFR: Estimated Glomerular Filtration Rate
ESRD: End-Stage Renal Disease
GERD: Gastroesophageal Reflux Disease
GI: Gastrointestinal
H2B: H2 Blocker
HR: Hazard Ratio
ICD-CM: International Classification of Disease-Clinical Modification
NHI: National Health Insurance
NHIRD: National Health Insurance Research Database
NSAIDs: Non-Steroid Anti-Inflammatory Drugs
NSARF: National Taiwan University Hospital Study Group on Acute Renal Failure
OTC: Over-The-Counter
PPIs: Proton Pump Inhibitors
RAAS: Renin-Angiotensin-Aldosterone System
RR: Relative Risk
