# Supplementary figures and images for: Accelerated risk of renal disease progression in pre-ESRD patients with proton pump inhibitors use: a nationwide population-based study
Source: BMC Nephrol. 2024 Dec 23;25:469. doi: 10.1186/s12882-024-03867-6 (PMC11667990; doi:10.1186/s12882-024-03867-6)

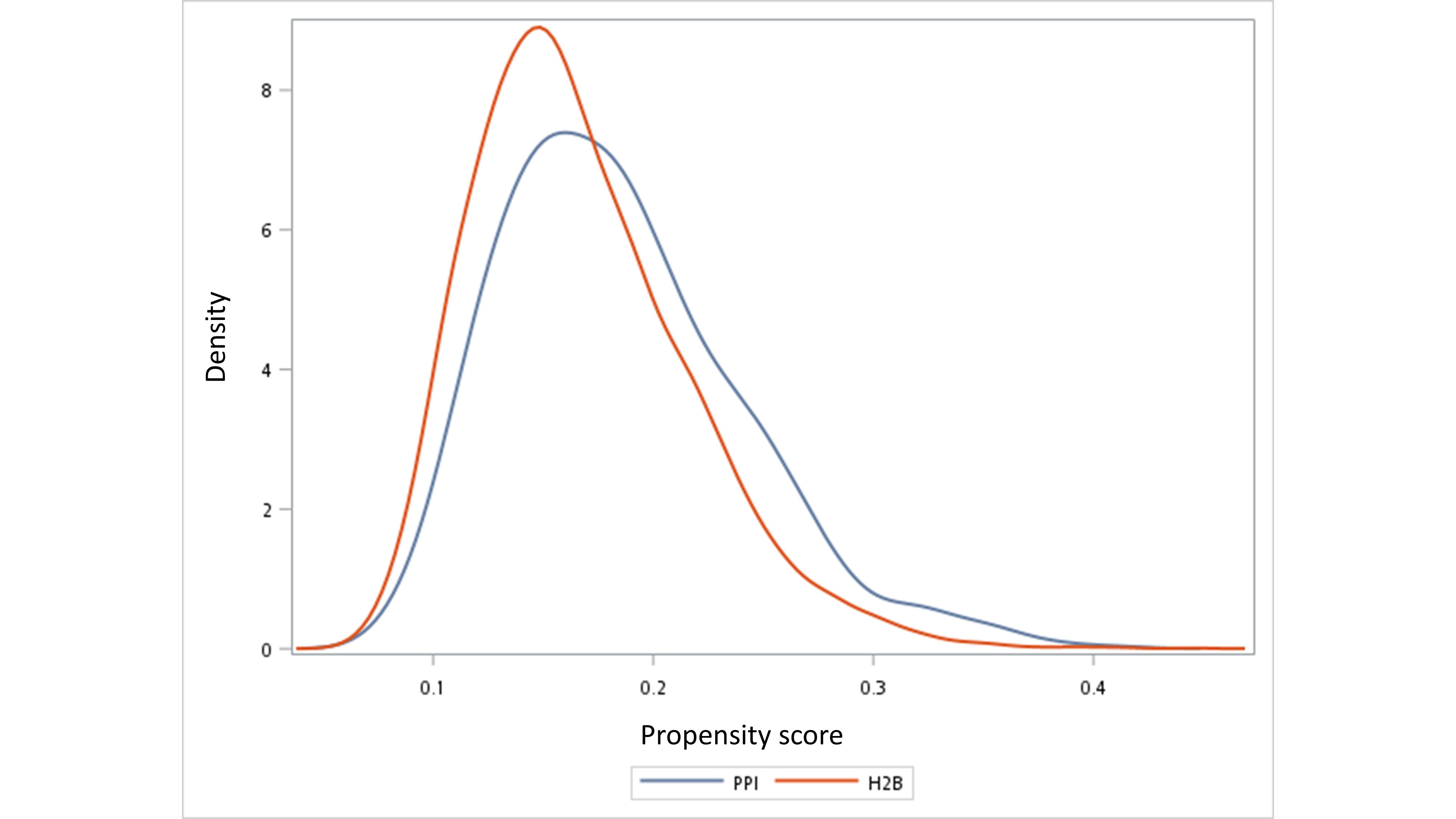

Supplement: Supplementary file 1 — Supplementary Material 1 [file 12882_2024_3867_MOESM1_ESM.jpg]

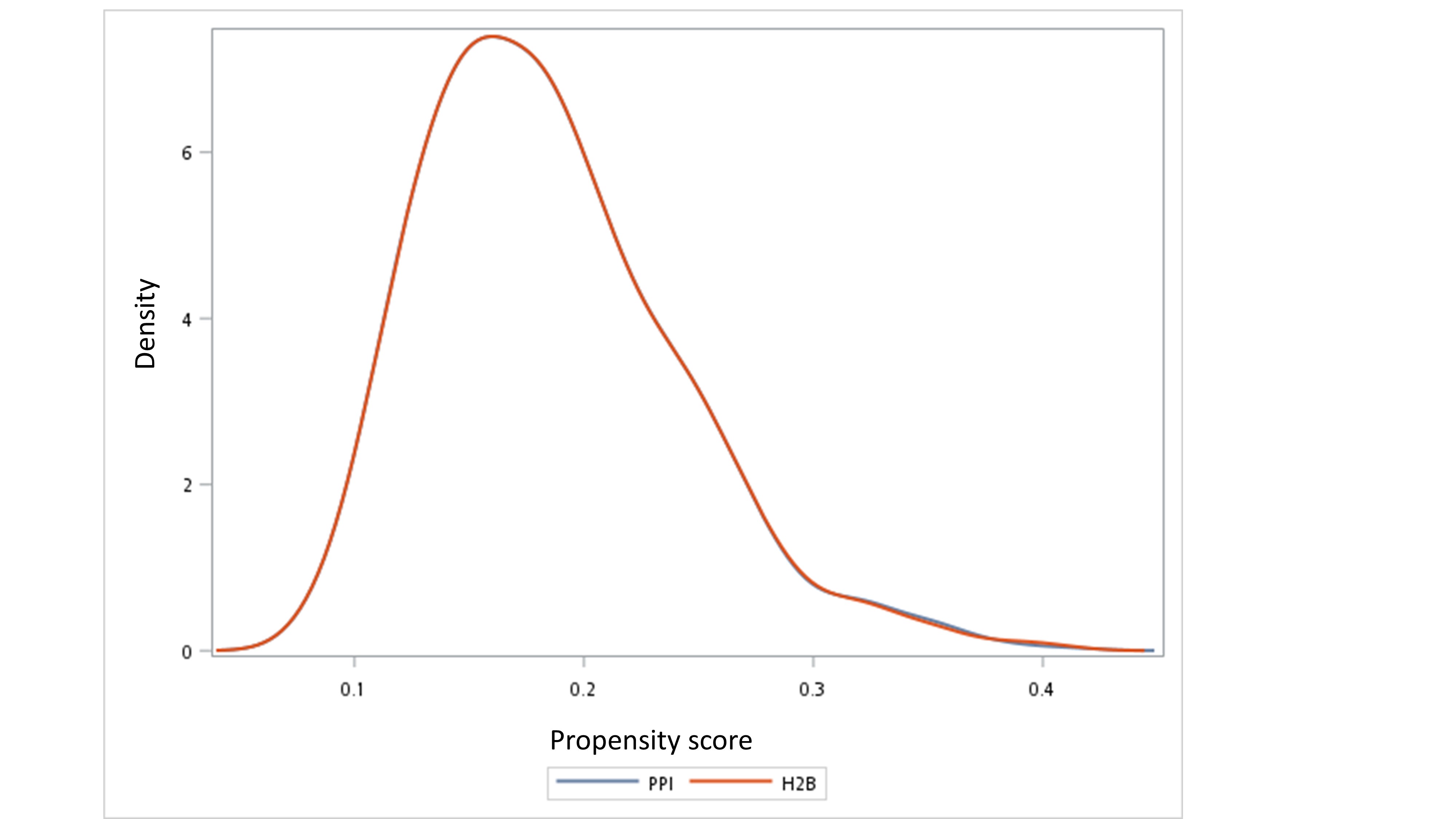

Supplement: Supplementary file 2 — Supplementary Material 2 [file 12882_2024_3867_MOESM2_ESM.jpg]

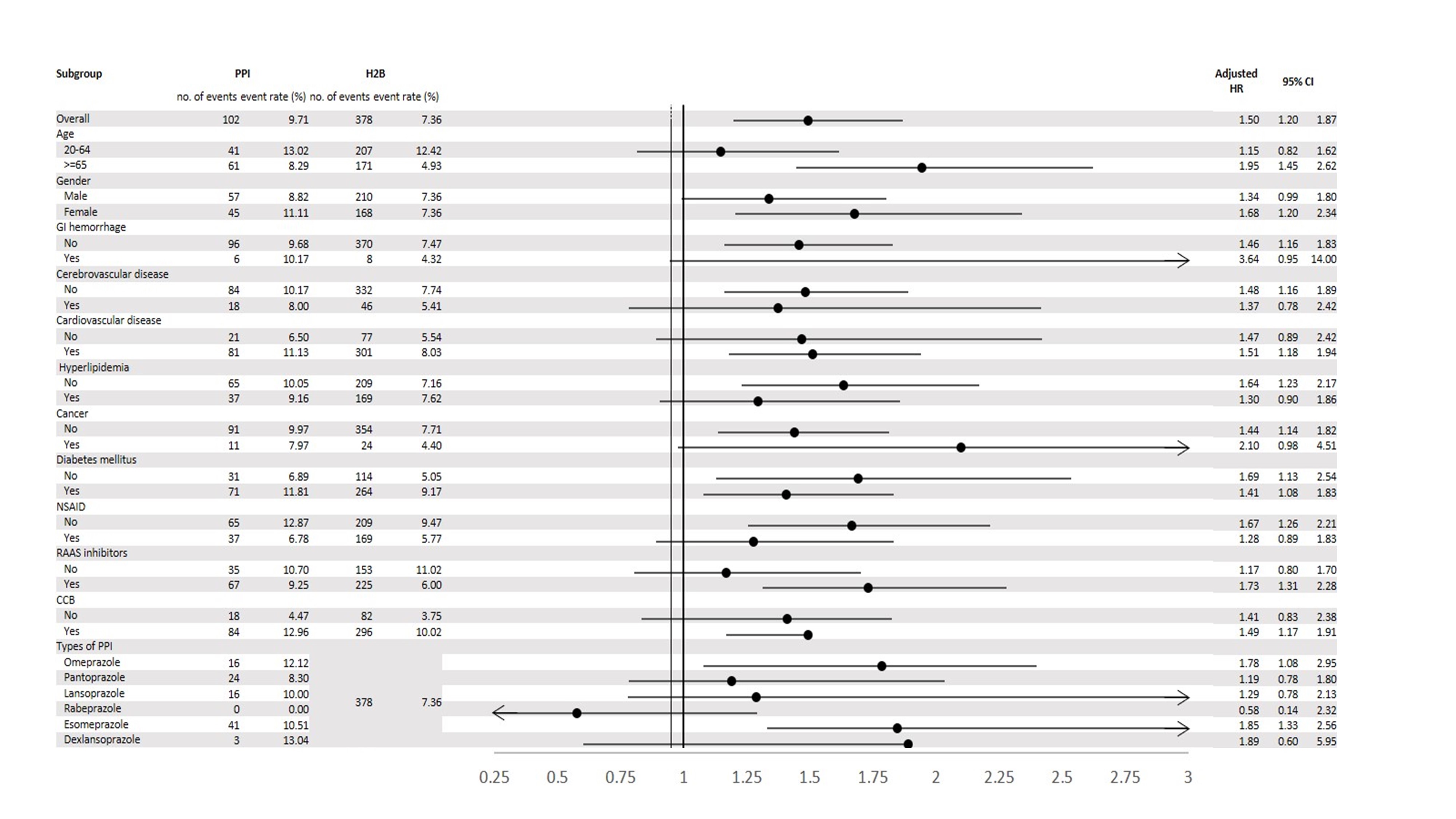

Supplement: Supplementary file 3 — Supplementary Material 3 [file 12882_2024_3867_MOESM3_ESM.jpg]

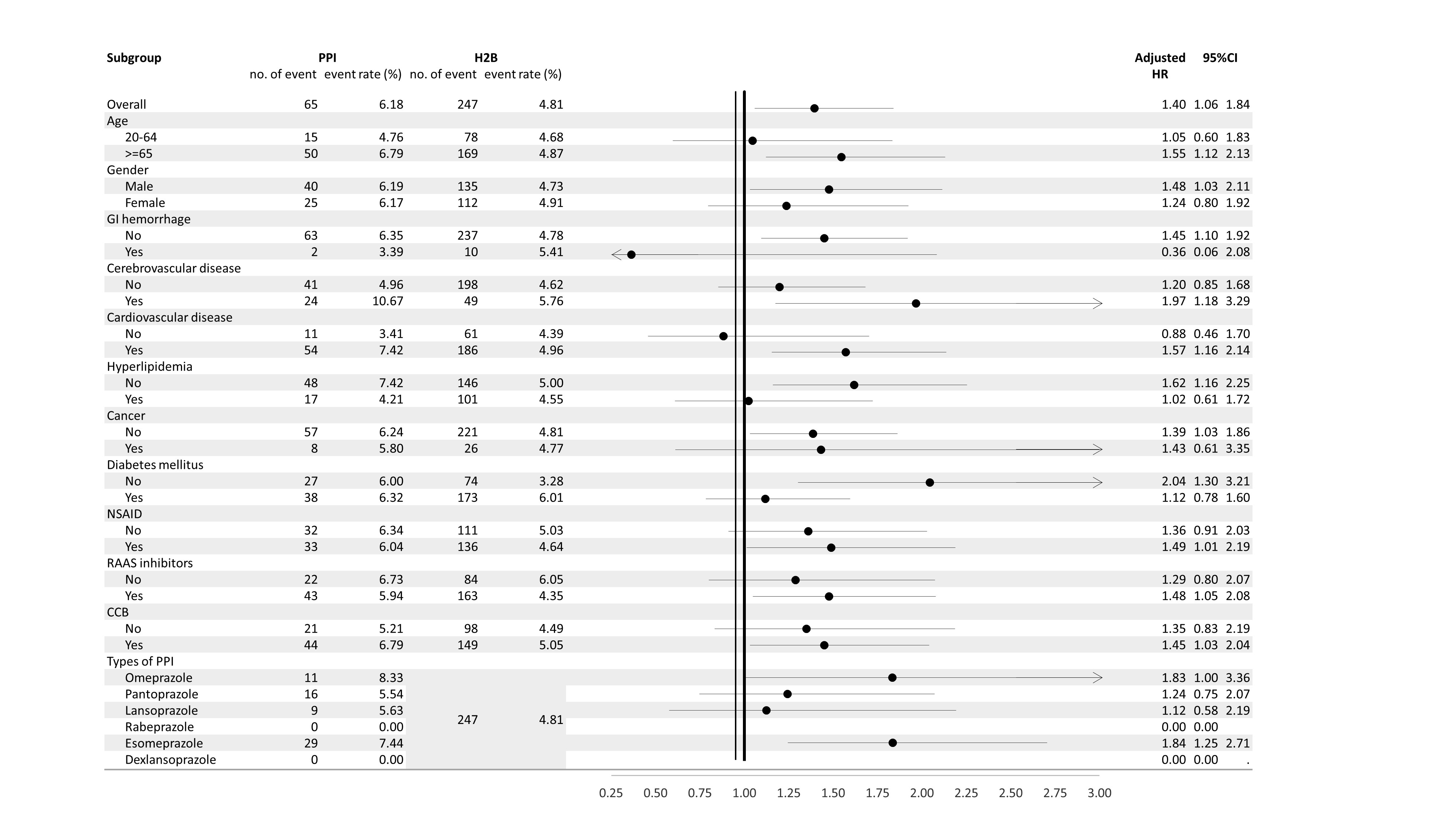

Supplement: Supplementary file 4 — Supplementary Material 4 [file 12882_2024_3867_MOESM4_ESM.jpg]

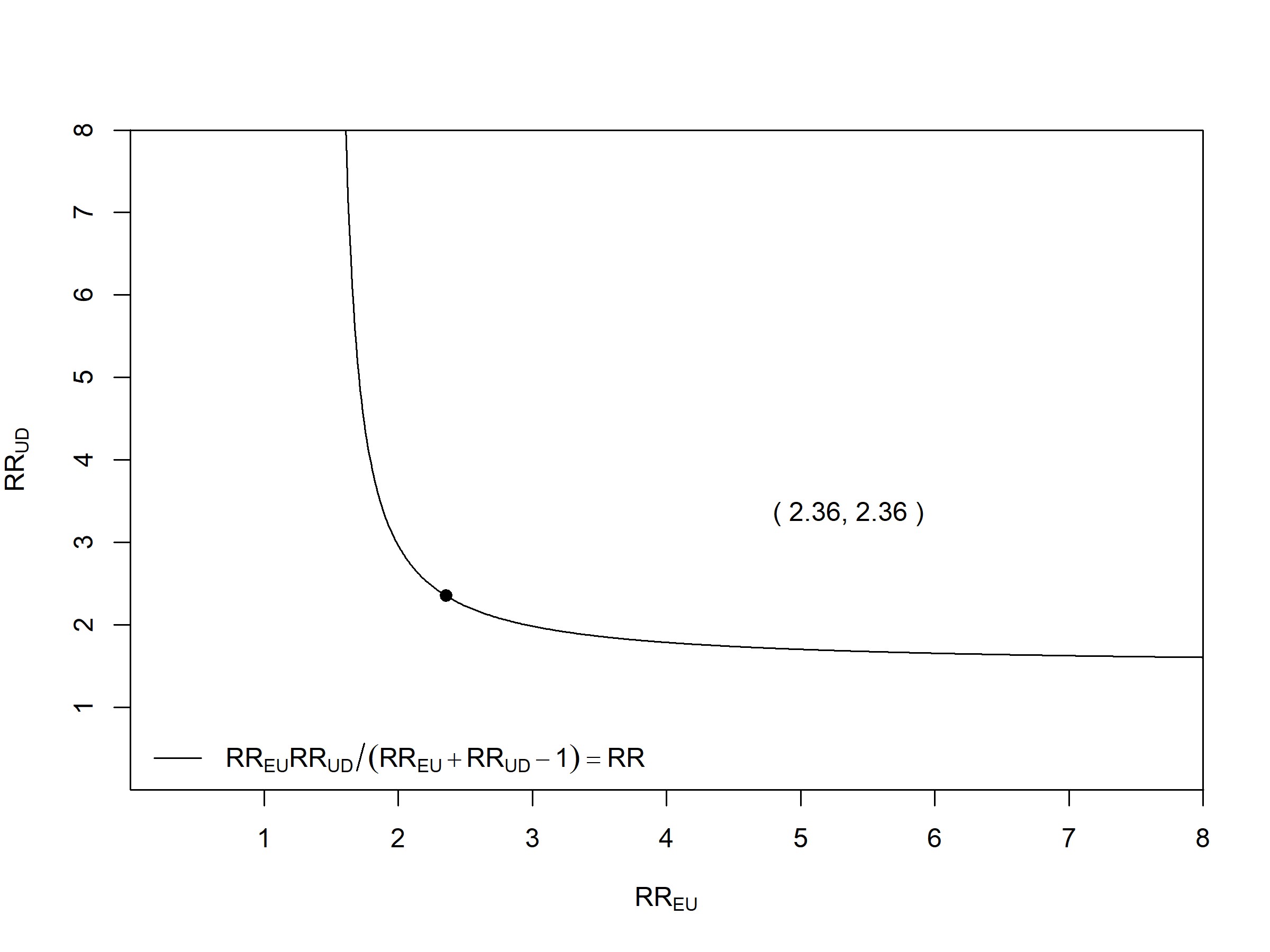

Supplement: Supplementary file 5 — Supplementary Material 5 [file 12882_2024_3867_MOESM5_ESM.jpg]
