# Supplementary material for: Accelerated risk of renal disease progression in pre-ESRD patients with proton pump inhibitors use: a nationwide population-based study
Source: BMC Nephrol. 2024 Dec 23;25:469. doi: 10.1186/s12882-024-03867-6 (PMC11667990; doi:10.1186/s12882-024-03867-6)
Supplement: Supplementary file 6 — Supplementary Material 6 [file 12882_2024_3867_MOESM6_ESM.docx]

Table S1. Reimbursement codes of the Pre-ESRD program

| **Drug**  **number** | **Benefit package** |
| --- | --- |
| **P3402C** | Management and care for new case |
| **P3403C** | Complete follow-up health education and care |
| **P3404C** | Annual evaluation |
| **P3405C** | Data processing for termination |
| **P3406C** | CKD Stage 3b and 4 patients of care incentive pay |
| **P3407C** | CKD Stage 5 patients of care incentive pay |
| **P3408C** | Patients with proteinuria of care incentive pay |
| **P3409C** | Continuous care incentive pay |
