# Supplementary material for: Accelerated risk of renal disease progression in pre-ESRD patients with proton pump inhibitors use: a nationwide population-based study
Source: BMC Nephrol. 2024 Dec 23;25:469. doi: 10.1186/s12882-024-03867-6 (PMC11667990; doi:10.1186/s12882-024-03867-6)
Supplement: Supplementary file 7 — Supplementary Material 7 [file 12882_2024_3867_MOESM7_ESM.docx]

Table S2. Diagnostic ICD-9-CM and ICD-10 codes for comorbidities

| Disease | ICD-9-CM code | ICD-10 code |
| --- | --- | --- |
| Gastro-esophageal reflux disease  (GERD) | 53011, 53081 | K21, K210, K219 |
| Gastrointestinal hemorrhage | 578 | K922 |
| Peptic ulcer disease (PUD) | 5302, 531–534 | Z8711 |
| Esophageal varices |  | I85, I850, I8500–I8501 |
| *Helicobacter pylori* (HP) infection | 04186 | B9681 |
| Cerebrovascular disease (CVA) | 430–438 | I60.00–I69.998 |
| Peripheral artery disease (PAD) | 440.0, 440.2–440.3, 440.8–440.9,  443, 444.0, 444.22, 444.8, 447.8–  447.9 | I70.0, I70.2, I70.9, I70.3, I70.8,  I75.89, I70.9, I73.0, I73.1,  I73.8, I73.9, I79.1, I79.8,  I74.01, I74.09, I74.3, I74.4,  I74.5, I74.8, I74.9, I77.9, I70.0, 41, 045, 047, 049, 04B, 04C,  04H, 04J, 04L, 04N, 04P, 04Q,  04R, 04S, 04U, 04V, 04W |
| Cardiovascular disease (CVD) | 401–414, 428, 42731 | I50.1–I50.9  I20.0–I25.9 |
| Hyperlipidemia | 272.0–272.4 | E78.0–E78.9 |
| Hypertension (HTN) | 401–405 | I10–I15.9 |
| Diabetes mellitus (DM) | 250 | E08.00–E13.9 |
| Chronic obstructive pulmonary  disease (COPD) | 490–491, 495–496 | J44.9 |
| Dementia | 290.0–290.4, 294.1, 331.0–331.2,  331.82 | F03, F039, F0390-391 |
| Cancer | 140-208 | C00.0–C96.Z |
| Viral hepatitis | 070 | B17, B178-179, B180-182,  B188-189, B19, B190-191 |
