# Supplementary material for: Accelerated risk of renal disease progression in pre-ESRD patients with proton pump inhibitors use: a nationwide population-based study
Source: BMC Nephrol. 2024 Dec 23;25:469. doi: 10.1186/s12882-024-03867-6 (PMC11667990; doi:10.1186/s12882-024-03867-6)
Supplement: Supplementary file 8 — Supplementary Material 8 [file 12882_2024_3867_MOESM8_ESM.docx]

Table S3. Baseline propensity score-matched Pre-ESRD subject characteristics

| **H2B users** | | | **PPI users** | | **P Value** |
| --- | --- | --- | --- | --- | --- |
|  | (n=1051) | | (n=1051) | |  |
| **Age**, mean (SD) y | 70.4 | (13.6) | 71 | (12.9) | 0.3328 |
| **Male**, n (%) | 644 | (61.3) | 646 | (61.5) | 0.9286 |
| **Comorbidities**, n (%) |  |  |  |  |  |
| GERD | 119 | (11.3) | 107 | (10.2) | 0.3981 |
| GI hemorrhage | 56 | (5.3) | 59 | (5.6) | 0.7736 |
| Peptic ulcer disease | 146 | (13.9) | 164 | (15.6) | 0.2682 |
| HP infection | 7 | (0.7) | 7 | (0.7) | 1.0000 |
| Cerebrovascular disease | 200 | (19.0) | 225 | (21.4) | 0.1746 |
| Peripheral artery disease | 51 | (4.9) | 56 | (5.3) | 0.6198 |
| Cardiovascular disease | 721 | (68.6) | 728 | (69.3) | 0.7415 |
| Obesity |  |  |  | 7 | 0.0584 |
| Hyperlipidemia | 391 | (37.2) | 404 | (38.4) | 0.5587 |
| Hypertension | 871 | (82.9) | 877 | (83.4) | 0.7266 |
| Diabetes mellitus | 606 | (57.7) | 601 | (57.2) | 0.8254 |
| COPD | 118 | (11.2) | 129 | (12.3) | 0.4562 |
| Dementia | 60 | (5.7) | 68 | (6.5) | 0.4656 |
| Cancer | 140 | (13.3) | 138 | (13.1) | 0.8975 |
| Viral hepatitis | 50 | (4.8) | 48 | (4.6) | 0.8361 |
| HIV infections | 0 | (0.0) | 0 | (0.0) | . |
| **Medication history**, n (%) |  |  |  |  |  |
| NSAIDs | 548 | (52.1) | 546 | (52.0) | 0.9304 |
| RAAS inhibitors | 715 | (68.0) | 724 | (68.9) | 0.6727 |
| Calcineurin inhibitors | 9 | (0.9) | 8 | (0.8) | 0.8076 |
| Diuretics | 595 | (56.6) | 582 | (55.4) | 0.5679 |
| Antivirals | 13 | (1.2) | 15 | (1.4) | 0.7036 |
| Antibiotics | 459 | (43.7) | 446 | (42.4) | 0.5669 |
| CCBs | 639 | (60.8) | 648 | (61.7) | 0.6870 |
| β-blockers | 482 | (45.9) | 473 | (45.0) | 0.6934 |
| Antithrombotics | 546 | (52.0) | 547 | (52.0) | 0.9652 |
| Statins | 391 | (37.2) | 404 | (38.4) | 0.5587 |

Abbreviations as in Table 1.
