# Supplementary material for: Accelerated risk of renal disease progression in pre-ESRD patients with proton pump inhibitors use: a nationwide population-based study
Source: BMC Nephrol. 2024 Dec 23;25:469. doi: 10.1186/s12882-024-03867-6 (PMC11667990; doi:10.1186/s12882-024-03867-6)
Supplement: Supplementary file 9 — Supplementary Material 9 [file 12882_2024_3867_MOESM9_ESM.docx]

Table S4. Baseline characteristics of Nonusers /H2B users

|  | Nonusers  (n=77,243) | | H2B users  (n=5,138) | |
| --- | --- | --- | --- | --- |
| **Age**, **mean (SD) y** | 67.9 | (13.8) | 69.7 | (13.3) |
| **Male**, **n (%)** | 46,050 | (59.6) | 2,855 | (55.6) |
| **Comorbidities**, **n (%)** |  |  |  |  |
| GERD | 3,647 | (4.7) | 441 | (8.6) |
| GI hemorrhage | 2,119 | (2.7) | 185 | (3.6) |
| Peptic ulcer disease | 7,116 | (9.2) | 798 | (15.5) |
| HP infection | 230 | (0.3) | 30 | (0.6) |
| Cerebrovascular disease | 12,162 | (15.7) | 850 | (16.5) |
| Peripheral artery disease | 3,296 | (4.3) | 289 | (5.6) |
| Cardiovascular disease | 55,556 | (71.9) | 3,747 | (72.9) |
| Hyperlipidemia | 33,886 | (43.9) | 2,218 | (43.2) |
| Hypertension | 63,166 | (81.8) | 4,244 | (82.6) |
| Diabetes mellitus | 42,085 | (54.5) | 2,879 | (56.0) |
| COPD | 6,959 | (9.0) | 627 | (12.2) |
| Dementia | 3,355 | (4.3) | 273 | (5.3) |
| Cancer | 6,867 | (8.9) | 545 | (10.6) |
| Viral hepatitis | 3,086 | (4.0) | 231 | (4.5) |
| **Medication history, n (%)** |  |  |  |  |
| NSAIDs | 35,670 | (46.2) | 2,930 | (57.0) |
| RAAS inhibitors | 57,265 | (74.1) | 3,749 | (73.0) |
| Calcineurin inhibitors | 270 | (0.3) | 27 | (0.5) |
| Diuretics | 31,931 | (41.3) | 2,416 | (47.0) |
| Antivirals | 939 | (1.2) | 72 | (1.4) |
| Antibiotics | 26,912 | (34.8) | 2,188 | (42.6) |
| CCBs | 43,394 | (56.2) | 2,953 | (57.5) |
| β-blockers | 32,704 | (42.3) | 2,239 | (43.6) |
| Antithrombotics | 37,712 | (48.8) | 2,704 | (52.6) |
| Statins | 32,825 | (42.5) | 2,124 | (41.3) |

H2B, histamine H2-blockers; PPI, proton pump inhibitor; SD, standard deviation; GERD, gastroesophageal reflux disease; GI, gastrointestinal; HP, Helicobacter pylori; COPD, chronic obstructive pulmonary disease; NSAIDs, non-steroid anti-inflammatory drugs; RAAS, renin-angiotensin-aldosterone system; CCBs, calcium channel blockers; ESRD; end-stage renal disease.
